# AlphaFold-Predicted Structures of KCTD Proteins Unravel Previously Undetected Relationships among the Members of the Family

**DOI:** 10.3390/biom11121862

**Published:** 2021-12-10

**Authors:** Luciana Esposito, Nicole Balasco, Giovanni Smaldone, Rita Berisio, Alessia Ruggiero, Luigi Vitagliano

**Affiliations:** 1 Institute of Biostructures and Bioimaging, CNR, Via Mezzocannone 16, 80134 Naples, Italy; nicole.balasco@unina.it (N.B.); rita.berisio@cnr.it (R.B.); alessia.ruggiero@cnr.it (A.R.); 2 IRCCS SDN, Napoli, Via E. Gianturco 113, 80143 Naples, Italy; giovanni.smaldone@synlab.it

**Keywords:** protein structure predictions, structure-based evolutionary trees, protein structure function

## Abstract

One of the most striking features of KCTD proteins is their involvement in apparently unrelated yet fundamental physio-pathological processes. Unfortunately, comprehensive structure–function relationships for this protein family have been hampered by the scarcity of the structural data available. This scenario is rapidly changing due to the release of the protein three-dimensional models predicted by AlphaFold (AF). Here, we exploited the structural information contained in the AF database to gain insights into the relationships among the members of the KCTD family with the aim of facilitating the definition of the structural and molecular basis of key roles that these proteins play in many biological processes. The most important finding that emerged from this investigation is the discovery that, in addition to the BTB domain, the vast majority of these proteins also share a structurally similar domain in the C-terminal region despite the absence of general sequence similarities detectable in this region. Using this domain as reference, we generated a novel and comprehensive structure-based pseudo-phylogenetic tree that unraveled previously undetected similarities among the protein family. In particular, we generated a new clustering of the KCTD proteins that will represent a solid ground for interpreting their many functions.

## 1. Introduction

The KCTD family (proteins containing the K-potassium Channel Tetramerization Domain) comprises twenty-five members (KCTD1-21, SHKBP1, TNFBP1, KCNRG, and BTBD10) involved in diversified yet fundamental physio-pathological processes. Their emerging role is clearly evident from the analysis of selected studies published in the last decade [1,2,3,4,5]. One of the most striking and puzzling features of these proteins is their involvement in apparently unrelated processes. KCTDs were initially identified as key factors of neurodevelopmental and neuropsychiatric disorders ([6] and references therein). The role of these proteins has been clearly assessed in neurocognitive disorders (KCTD3), epilepsy (KCTD7), bipolar disorder (KCTD12), autism and schizophrenia (KCTD13), and movement disorders (KCTD17). Moreover, almost all of the members of the family have been associated with the insurgence and the progression of different types of cancers ([7] and references therein), including leukemia, medulloblastoma, hepatocarcinoma, and breast, pancreatic, and colorectal cancer. In addition, KCTD proteins are also implicated into the insurgence of genetically inherited diseases such as the scalp–ear–nipple syndrome [8,9] and obesity/adipogenesis [10,11].

The founding feature of this protein family is the presence in all of the members of a conserved BTB (Broad complex, Tramtrak and Bric-a-brac) domain (also denoted as POZ—poxvirus zinc finger—or T1) [12]. In its canonical function, the BTB domain is involved in both homo- and hetero-oligomerizations [13]. In KCTD proteins, the pentameric association is the prevalent functional oligomerization state [14,15,16,17], although their BTB domains have shown a remarkable versatility in the formation of assemblies formed by a variable number of polypeptide chains [15,18]. These observations have led to the suggestion that the primary role of the BTB domain in KCTDs is in partnership formation rather than in dictating oligomerization. Indeed, key interactions of KCTD proteins that are established with partners such as cullin 3 [17,19,20], the transcription factor AP-2α [21], and the receptor GABAB2 [5,22] are mediated by the BTB domain. The BTB domain is located in the N-terminal region of KCTD protein and, with very few exceptions, it is preceded in the sequence by small, and likely unstructured, protein stretches. In the various members of the family, the BTB domain is followed by regions whose sequences are globally unrelated among KCTDs. Indeed, sequence similarities in the C-terminal regions can be detected in selected groups of KCTD proteins made by two or three members. Based on the alignments of the BTB domains, Skoblov et al. [23] generated an influential divergence tree of KCTD proteins in which proteins presenting sequence similarities in the C-terminal region were grouped in the same clade (Figure S1). Although, over the years, modifications of this tree have been proposed [6,15,24], it is considered a reliable classification of KCTD sequences and, presumably, also representative of structure similarities.

Despite the recognized role of these proteins in key biological processes, a full understanding of their biochemical role is far from being achieved. Although, for some of the members, a clear role as substrate adaptor in cullin-ring ligases in protein ubiquitination/degradation has been established [23], their involvement in unrelated physio-pathological processes remains an intriguing but essentially unsolved issue. It is important to note that it has been pointed out that even individual members of the family can play roles in apparently distant contexts. This observation has suggested that these KCTDs are likely involved in some basic, hitherto undiscovered, biochemical activities [25].

It is commonly believed that the poor and fragmentary structural data available for these proteins has hampered insightful definitions of structure–function relationships in these proteins. Indeed, the three-dimensional structure of the BTB domain has been reported only for a limited, although significant, number of KCTD proteins, whereas the full-length structure has only been reported for two members of the family (Table 1). Moreover, sporadic information is available regarding the folded domains present in the diversified C-terminal regions (Table 1). This scenario is going to rapidly change if the structures predicted by AlphaFold [26,27] and deposited in the AlphaFold Protein Structure Database (AF Database—https://alphafold.ebi.ac.uk/ accessed on 1 November 2021) are considered. Indeed, reliable three-dimensional structures have been reported for all KCTD proteins. Here, we globally analyzed these models by highlighting analogies and differences among the members of the family. We also assessed the accuracy of these models by comparing the experimental versus the predicted structures, for the proteins whose structure had been previously crystallographically determined. These analyses clearly indicated that, despite undetectable sequence analogies, the C-terminal region of the vast majority of KCTD proteins share a common structural domain (KCTD-CTD). Using this domain as a reference, we generated a novel comprehensive structure-based divergence tree that unraveled previously undetected similarities among the protein family. We believe that the present findings will facilitate the definition of the thus far elusive structure–function relationships of these proteins.

## 2. Materials and Methods

### 2.1. Three-Dimensional Structures of KCTD Proteins and Their Analysis

The predicted three-dimensional structures of the KCTD proteins characterized here were retrieved from the AlphaFold Protein Structure Database (AF Database) created by DeepMind and EMBL’s European Bioinformatics Institute (EMBL-EBI) (https://alphafold.ebi.ac.uk accessed on 21/08/2021). As in the database for the protein KCTD11, the model of the truncated form of the protein (Uniprot code Q693B1-1), in which the N-terminal region of the BTB domain is missing, was reported, we generated a three-dimensional model for the long variant (Uniprot code Q693B1-2), which is the functional form of the protein [28], using the Colab server (https://colab.research.google.com/github/sokrypton/ColabFold/blob/main/AlphaFold2.ipynb accessed on 15/09/2021). This server predicts protein structures starting from their sequences using a slightly simplified version of AlphaFold v2.0 that does not consider existing structural templates. The Colab server was also used to generate an unbiased template-free model of the KCTD1 protein. The reliability of the AF predictions was assessed by the Local Distance Difference Test (LDDT) score reported for each structure of the AlphaFold Database.

The experimental crystallographic structures of KCTD proteins were retrieved from the Protein Data Bank (https://www.rcsb.org accessed on 15/10/2021) using the keyword KCTD to search the database. These analyses were integrated using the BLAST server (https://blast.ncbi.nlm.nih.gov/Blast.cgi accessed on 21/08/2021) and the sequence of KCTDs as query. Both experimental and predicted structures were inspected by molecular graphics and using available software such as PDBSUM [29] and Dali (http://ekhidna2.biocenter.helsinki.fi accessed on 1 November 2021) [30,31] to identify secondary structure elements and domain boundaries.

### 2.2. Comparison of the Three-Dimensional Structures

Extensive comparative analysis of the structures of the KCTD proteins was performed using Dali (http://ekhidna2.biocenter.helsinki.fi/dali/ accessed on 1 November 2021). In particular, pairwise comparisons were performed between PDB structures. Moreover, we selected the C-terminal domains of the AF models and structurally aligned every pair of domain structure by using the DALI distance matrix alignment method and scoring function [32], as implemented in the Dali web server. The Dali server was also used to search the PDB for structural analogs of the predicted KCTD structures.

### 2.3. Generation of Structural Tree

After the generation of the pairwise structural similarity matrix by reporting the Dali Z-score, the structures could be grouped into clusters, and the relationships among various clusters could be showed by a dendrogram (a diagram representing a tree). The Dali server provided a dendrogram of the structures by using hierarchical agglomerative clustering based on the average linkage method applied to the structural similarity matrix. The structural dendrogram in Newick format, returned by Dali all-against-all comparison, was read by the PHY·FI server (https://services.birc.au.dk/phyfi/go.py accessed on 1 November 2021) [33] to obtain the graphical representation.

In addition to providing the pseudo-phylogenetic tree, Dali also performed a multidimensional scaling correspondence analysis of the Z-scores. The results of the projection of the structures on the first two eigenvectors are plotted in the correspondence analysis plot where the most similar structural neighborhoods are placed near each other.

## 3. Results

### 3.1. KCTDs in the PDB: The State of the Art

A survey of the PDB (October 2021 release) indicates that twenty-one distinct structures of KCTD proteins have been hitherto reported in the database (Table 1). Among these, only two cover a significant portion of corresponding proteins and may be considered as representative of the structures of the full-length proteins. The structure of KCTD5 (PDB entries 3DRX and 3DRY), the first reported full-length structure for a member of the family, presents a pentameric oligomerization state whose overall interdomain association depends on the salt conditions [16]. The full-length structure of KCTD1 (PDB entry 6S4L—doi 10.2210/pdb6S4L/pdb) is also a pentamer and covers, with the exception of the N- and C-terminal tails, the entire protein. All of the other structures correspond to the individual domains. The structure of the BTB domain, as an isolated entity, has been reported for KCTD1 (PDB entries 5BXD and 5BXB), KCTD5 (PDB entry 3DRZ), KCTD10 (5FTA), KCTD13 (4UIJ), KCTD16 (5A15, 6OCR, 6I0Q, and 6OCT), KCTD17 (5A6R), and SHKBP1 (4CRH). These structures have highlighted the versatility of this domain [15], which was also corroborated by molecular dynamics simulations [18], to oligomerize in different oligomeric states that include monomeric, tetrameric, open/close pentameric, and hexameric assemblies [15,17,34]. This domain has also been characterized in complex with a peptide of the GABAB2 receptor (6OCP and 6M8R) [22,35].

The other PDB structures correspond to the folded domain occurring in the C-terminal regions of the closely related proteins KCTD8 (6G57), KCTD12 (6QZL), and KCTD16 (6QB7). This domain, also denoted in the literature as the H1 domain, has also been reported in complex with the Gβ1γ2 subunits (6M8S) [22].

**Table 1:** Structures of KCTD proteins in the PDB and their similarity with the corresponding AlphaFold models. If multiple crystal structures of the same protein/domain were present, the one determined at the highest resolution was used for calculation of RMSD values.

Protein	Domain/Complex	PDB Code(s)/References	Resolution(s) (Å)	RMSD (Å) (#) ^1^
KCTD1	BTB	5BXD/5BXB [17]	1.8/2.2	0.6 (103)/0.4 (103)
	Full-length	6S4L	2.4	0.8 (205)
KCTD5	BTB	3DRZ [16]	1.9	0.8 (102)
	Full-length	3DRX/3DRY [16]	3.1/3.3	2.1 (163)/2.4(133)
KCTD8	CTD	6G57	2.8	1.8 (88)
KCTD9	BTB	5BXH [17]	2.8	0.7 (97)
KCTD10	BTB	5FTA [15]	2.6	0.4 (96)
KCTD12	CTD	6QZL	2.0	0.7 (100)
	CTD-Gβ1γ2	6M8S [22]	3.7	0.8 (103)
KCTD13	BTB	4UIJ [15]	2.7	0.3 (102)
KCTD16	BTB	5A15/6OCR/6I0Q/6OCT [15,34,35]	2.8/2.3/2.3	0.5 (91)/0.4 (90)/0.7 (95)/0.5 (91)
	CTD	6QB7	2.2	0.7 (110)
	BTB-GABA_B2 pept_	6OCP/6M8R [22,35]	2.3/3.2	0.5 (91)/0.5 (93)
	BTB	5A6R [15]	2.8	0.7 (101)
	BTB	4CRH [15]	1.7	1.0 (90)

^1^ This represents the number of the superimposed residues.

### 3.2. AlphaFold Versus PDB KCTD Structures

**Figure 1:**
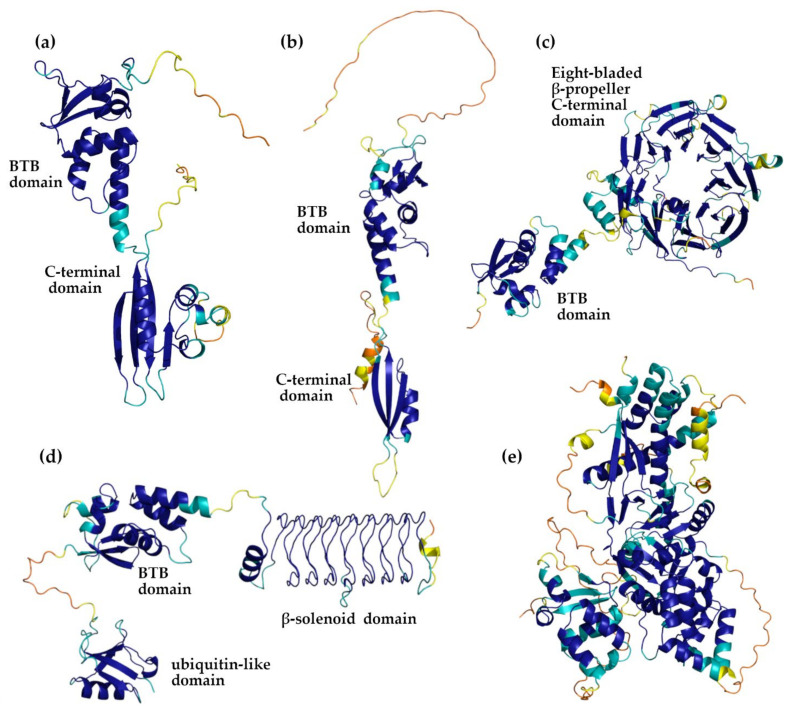
Representative AF-predicted models of the following KCTD proteins: KCTD1 (**a**), KCTD5 (**b**), KCTD3 (**c**), KCTD9 (**d**), and KCTD19 (**e**). The color code of the cartoons follows that used by AlphaFold to report the reliability of the models. Blue, cyan, orange, and yellow protein regions correspond to very high (LDDT > 90), high (90 > LDDT > 70), low (70 > LDDT > 50), and very low (LDDT < 50) model confidence, respectively.

**Figure 2:**
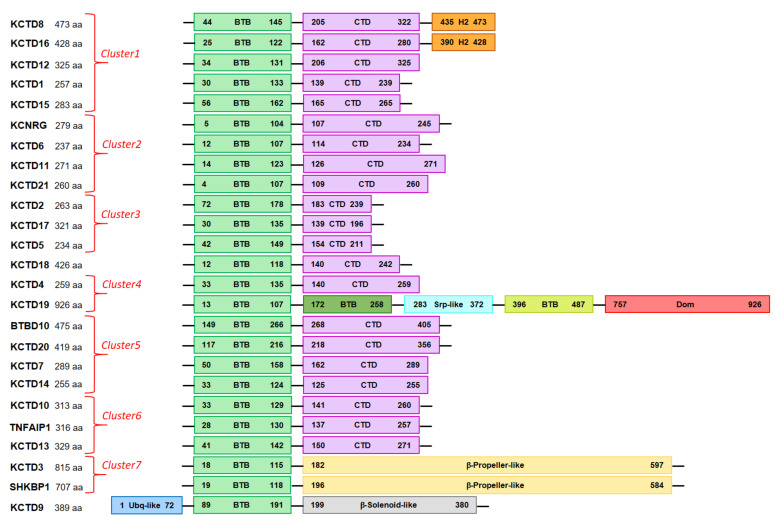
Schematic representation of protein domain organization. For the KCTD10, KCTD13, and TNFAIP1 proteins, AF predicts that a potential β-strand of the flexible C-terminal region could join the β-sheet of the CTD domain. The Dom domain of KCTD19 presents some similarity to the TAFH domain.

The analysis of three-dimensional structures of the human KCTD proteins predicted by AF indicates that they present significant variations among the members of the family that, however, operate on a common theme. As expected on the basis of their sequences, all AF models of KCTD proteins present a BTB domain which is canonically constituted by a single β-sheet surrounded by five helices (Figure 1). In addition to this common element, all KCTD members present other folded regions that are occasionally accompanied by unstructured fragments characterized by a low confidence in the prediction (Table 2). In line with previous indications, in most of the KCTD proteins, in addition to the BTB, there is a single extra folded domain located at the C-terminus whose size ranges from 57 to 146 residues (Table 2 and Figure 1 and Figure 2). One exception to this general trend is observed for the proteins KCTD3/SHKBP1, which present much larger C-terminal folded domains that assume the structure of an eight-bladed β-propeller (Figure 1). Other examples include KCTD9, which exhibits two extra domains in addition to the BTB that belong to the ubiquitin-like and to the pentapeptide repeat family of β-solenoid classes (Figure 1 and Figure 2), and KCTD19, which presents a collapsed multidomain structure that contains three BTB domains (Figure S2). As anticipated above, in addition to the folded domains, several KCTD proteins also exhibit long unstructured portions that can behave as intrinsically disordered regions (Table 2).

The comparison of the AF-predicted structures with those experimentally determined and reported in the PDB clearly highlights the ability of this approach to correctly predict the structures of individual domains. Indeed, as shown in Table 1, most of the root mean square deviations (RMSD) of the predicted structures of the individual BTB domains versus the experimental structures range in the interval of 0.4–0.8 Å. It is worth noting that these values are close to the errors associated with experimental structures. A slightly higher value is detected for the BTB domain of SHKBP1 (1.0 Å), which is the only monomeric crystalline structure of the family hitherto reported. A similar scenario emerges for the evaluation of the folded domain present in the C-terminal region of the proteins. Again, the RSMD values fall in the interval of 0.7–0.8 Å. The only significant exception is represented by the C-terminal domain of KCTD8 (1.8 Å) that assumes a peculiar organization in the crystalline state (Figure S3a) that is radically different from the pentameric organization of the C-terminal domain observed in the close homologs KCTD12 and KCTD16 (Figure S3). It is worth mentioning that functional studies have predicted the presence of an additional domain (H2) in the C-terminal region of the sequence of KCTD8 and KCTD16. The inspection of the corresponding AF structures shows the presence of two rather isolated helices at the C-terminus of these two proteins, with a reliable prediction score (90 > LDDT > 70). Although speculative, this observation may suggest that the H2 domain is a sort of flexible modulator of the activity of these proteins.

The excellent prediction of the full-length structure of KCTD1 (RMSD of 0.8 Å) shows the ability of the AF approach to correctly determine the relative position of the two domains. Larger RMSD values are detected for the full-length structure of KCTD5 (2.1–2.4 Å). However, it should be noted that the global structure of this protein is somehow flexible, as demonstrated by its sensitivity to salt conditions and corroborated by molecular dynamics investigations [16,36].

It should be noted that the latest version of AlphaFold (AlfaFold2), which was used to generate the models here analyzed, considers the available experimental PDB structures that are used as templates. Although it has been shown that these templates have a marginal impact on the predictions [26], in principle, the excellent agreement between the prediction and the experimental structures detected here may be biased by the AlphaFold2 protocol. In order to assess the impact of the template on these findings, we ran predictions of the KCTD1 structure using the Colab server that uses the AlphaFold approach without employing any template in the predictive scheme (see Materials and Methods for details). The analyses of the structure predicted using this approach clearly indicate the overall correctness of the structure of the two domains of the protein. A significant increase is observed in the RMSD value computed on the entire structure that passed from 0.8 (AF model versus the experimental one) to 3.1 (Colab model versus the experimental one) Å. The structure of the two individual domains are, however, correctly predicted. Collectively, these findings indicate that the inclusion of the KCTD1 template in the AF approach only (slightly) affected the relative orientation of the two domains that constitute the protein (Figure S4).

### 3.3. Detecting Analogies among AlphaFold Structures of KCTD

As illustrated in the previous paragraph, the release of the AF-predicted structures grossly expanded the structural data available for KCTD proteins. A global visual inspection of these models also indicates that the folded domain occurring in the C-terminal region of these proteins has some common structural features. Indeed, in almost all structures, despite the significant size variability, this domain consists of a single β-sheet that is surrounded by few helices. On the basis of this observation, we undertook a quantitative comparison of these domains using the pairwise alignment tool of Dali (see Methods for details).

As shown in Table 3, in which the Z-score values that emerged from the Dali structural alignment are grouped according to the clades identified by Baranova and colleagues [23], the folded domains of the family member belonging to the same clade present high similarities, as demonstrated by the high Z-score values (also see Figure S5). This finding is not surprising, as these clades were defined according to the sequence homology of the C-terminal region [23]. Somewhat unexpected is the finding that significant structure analogies are frequently found among KCTD proteins belonging to different clades, despite the absence of significant sequence similarities.

A deeper inspection of the table unravels other interesting trends. The only member of the family whose C-terminal region does not display any structural similarities with the C-terminal regions of the other KCTD proteins is KCTD9, which presents a β-solenoid domain in this region (Figure 1). As expected on the basis of the peculiar structure of their C-terminal region, which is characterized by the presence of a large β-propeller, KCTD3 and SHKBP1 present remarkable similarities only between each other. However, a small but significant similarity (Z-score of 2.7) is detected between the C-terminal region of SHKBP1 and the corresponding region of KCTD21. As shown in Figure S6, the C-terminal domain of KCTD21 partially overlaps with one of the blades of the SHKBP1 β-propeller. Somewhat intriguing is the result that emerged from the search of this domain in KCTD19 that, as described above, has a peculiar structural organization that is characterized by a multidomain globular fold and the presence of three BTB domains (Figure 2). Indeed, small significant similarity in terms of Z-score values is detected between KCTD4, KCTD6, and BTBD10. Surprisingly, the region of KCTD19 that presents this similarity corresponds to the second BTB domain of the protein. As shown in Figure S7, this portion of the protein can be successfully fitted to both the BTB and the C-terminal domain of KCTD4.

Remarkable similarities in terms of pairwise Z-scores between KCTD proteins not belonging to the same previously reported clade are detected between: (a) members of the clades A and F, (b) KCNRG with KCTD6 and KCTD15, and (c) KCTD7 and KCTD14.

The detected structural analogy of the domain in the sequence region that follows the BTB domain of the vast majority of KCTD proteins suggests that also these portions of these proteins have a common origin. This new identified domain that presents a single β-sheet surrounded, depending on the specific KCTD protein, by two to five α-helices is thereafter denoted as the KCTD-CTD domain. Moreover, considering the absence of KCTD-CTD in KCTD9 and SHKBP1/KCTD3, we denote them as non-canonical KCTD proteins.

### 3.4. Structure-Based Pseudo-Phylogenetic Tree

**Figure 3:**
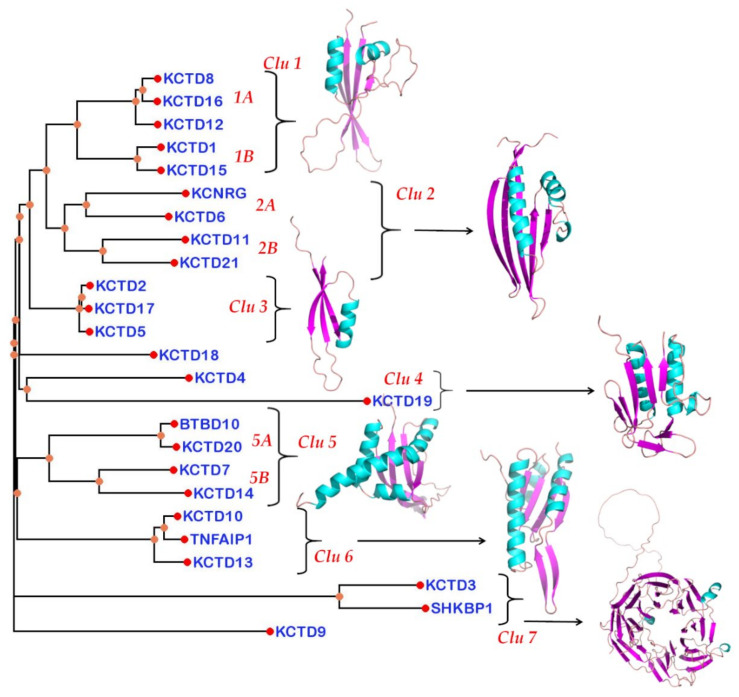
Dali dendrogram generated using the structure of the KCTD-CTD domain. Representative three-dimensional models of the folded domains present in the C-terminal region are also shown. Helices and β-sheets are shown in cyan and magenta, respectively. The cluster numbering is reported in red.

Once we assessed the reliability of the KCTD structures reported in the AF database and the presence of a conserved but diversified structural domain that follows the BTB domain, we decided to generate a structure-based phylogenetic-like tree by comparing the structures of the KCTD-CTD domain. The corresponding structural dendrogram, which was generated using the Dali package (see Methods for details), is reported in Figure 3. The dendrogram that emerged from this analysis highlights a clear clustering of the KCTD proteins. As expected on the basis of the results illustrated in the previous paragraph, KCTD9 and SHKBP1/KCTD3 are the proteins that are most distant from the other members of the family.

Notably, with the exception of KCTD18, all other canonical members of the family are clustered in groups that contain two or more members of the family (Figure 3). Moreover, some of the groups present some additional branching. Based on these observations, KCTD proteins are classified in clusters and sub-clusters. The first group (cluster 1) contains the proteins KCTD8, KCTD12 and KCTD16 (sub-cluster 1A) and KCTD1 and KCTD15 (sub-cluster 1B). The next group (cluster 2) comprises KCNRG and KCTD6 (sub-cluster 2A) and KCTD11 and KCTD21 (sub-cluster 2B). The upper clustering of the dendrogram is completed by cluster 3 that is made up of KCTD2, KCTD17, and KCTD5 (Figure 3). The upper portion of the diagram is completed by the isolated protein KCTD18 and by cluster 4 that embodies the distantly related KCTD4 and KCTD19. The lower portion of the tree includes clusters 5 and 6. Cluster 5 is articulated in the sub-clusters 5A (BTBD10 and KCTD20) and 5B (KCTD7 and KCTD14), whereas cluster 6 comprises KCTD10, TNFAIP1, and the slightly divergent KCTD13. Finally, in cluster 7, the two non-canonical KCTDs, KCTD3 and SHKBP1, are included.

This overall grouping is confirmed by the multidimensional scaling correspondence analysis of the Z-scores performed by Dali (Figure S8).

## 4. Discussion

Generalized and reliable predictions of three-dimensional protein structures from their sequences have been the holy grail of structural biology for decades. Indeed, despite the huge technical and methodological advances experienced in recent decades, the experimental determination of biomolecules of such complexity remains a challenging and lengthy task. As is universally agreed, the impact of AlphaFold predictions [26,27], whose accuracy often competes with that of experimental determinations, will certainly revolutionize the structural biology with implications that will affect distant fields of life sciences [37,38]. Here, we exploited the structural information contained in the AF database to gain insights into the relationships among the members of the KCTD family with the aim to facilitate the definition of the structure/molecular basis of their key roles in many biological processes.

The most important finding that emerged from the analysis is the discovery that, in addition to the BTB domain, the vast majority of these proteins share a structurally similar domain in the C-terminal region (KCTD-CTD) despite the general absence of sequence similarities detectable in these regions. In this scenario, the few exceptions (KCTD9, KCTD3, and SHKBP1), which present a different repertoire of domains, may be considered non-canonical members of the family. Nevertheless, the individual blades of the KCTD3/SHKBP1 β-propeller present some significant similarity with the CTD domain of KCTD21. Although this analogy may be coincidental, it can also be reminiscent of a very distant common origin of the β-propeller and the KCTD-CTD. Intermediate features are displayed by KCTD19 that presents a collapsed globular structure, in which three distinct BTB domains can be identified. Intriguingly, the third BTB domain of this protein has significant structural analogies with some CTD domains of the protein family (Table 3), although it is more similar to the other BTB domains. In structural terms, the BTB3 domain of KCTD19 is a sort of link between the BTB and the KCTD-CTD structures. Although this analogy may be due to a convergent evolution, it is intriguing to speculate that even the BTB and the CTD domain could share a common ancestor.

The finding that KCTD proteins, in addition to the BTB, also share a rather conserved CTD domain, whose structure is very likely to be correctly predicted by AF, prompted us to build a structure-based pseudo-phylogenetic tree that was generated by comparing the divergence of this domain throughout the family. This led to a new clustering of the KCTD proteins that also presents, along with analogies, significant differences when compared to the trees built on the basis of the BTB sequence comparisons. In general terms, the analysis of this pseudo-phylogenetic tree indicates that KCTD proteins belonging to different clusters may play important roles in cancer [7] or in neurodevelopmental and neuropsychiatric disorders [6], the most important diseases in which these proteins are involved. This may be the consequence of the adaptor function of some KCTDs that can recruit different substrates for ubiquitination in different physio-pathological contexts [6] or of other basic biochemical activities of these proteins yet to be identified. Nevertheless, novel clustering discriminates KCTD proteins on the basis of their ability to bind cullin 3. Indeed, independent experimental reports [15,17,19] have clearly demonstrated that members of cluster 1 (KCTD1/KCTD15 and KCTD8/KCTD12/KCTD16) are unable to bind cullin and, therefore, cannot function as adaptors of cullin-ring ligases. It is likely that the separation of these proteins from the nearby group of KCTD6/KCNRG/KCTD11/KCTD21, which do act as adaptors in the ubiquitination process, has been accompanied by a radical change of function of these proteins. Moreover, in this particular clustering, the analysis of the grouping of KCTD6, KCNRG, KCTD11, and KCTD21 indicates that these four proteins are generally related but separated in two divergent groups (KCTD6/KCNRG and KCTD11/KCTD21). Therefore, the KCASH functional subfamily of KCTD6/KCTD11/KCTD21 [39] should also include KCNRG as a member. Since the other three members of the group are able to downregulate HDAC [1,39], it would be of interest to verify whether KCNRG can also accomplish this function. Furthermore, the tree unravels the similarity of the pair KCTD20/BTBD10 with KCTD7/KCTD14. It also shows that the most divergent group within the canonical KCTDs is represented by KCTD10/KCTD13/TNFBP1, which are involved in the ubiquitination and degradation of small GTPases.

It is important to note that in the current AF database, only individual polypeptide chains are reported. As KCTD proteins generally operate as oligomers, likely pentamers, the ability of this predictive approach to provide information in spite of this important limitation is impressive. Obviously, the elucidation of the structure of KCTDs’ oligomeric states will hold important implications for their function. Our preliminary molecular modeling and dynamics studies indicate that most of the KCTD-CTD domains may form assemblies, such as those detected in the experimental structures of the CTD domains of KCTD5, KCTD1, KCTD12, and KCTD16, in which the association of the five monomers generates a channel that can be functionally important. Although further investigations are needed for a complete definition of the structural properties of these proteins, we believe that the AF database represents a treasury whose exploitation will revolutionize many areas of life sciences.

In conclusion, not only does this work give a structural interpretation of existing functional data on KCTD proteins but it also provides a new interpretative tool for the results that will emerge from future experiments.

## Figures and Tables

**Table 2:** Details of the KCTD AlphaFold models.

Protein	AlphaFold Code	Low Structured and/or Low Confident Regions ^1^	BTB Domain	CTD Domain
KCTD1	AF-Q719H9	1–17/242–257	A30-T133	P139-L239
KCTD2	AF-Q14681	1–71/239–263	R72-T178	V183-N239
KCTD3	AF-Q9Y597	1–17/139–181/598–815	E18-L115	- ^4^
KCTD4	AF-Q8WVF5	1–32/198–217	T33-L135	T140-K259
KCTD5	AF-Q9NXV2	1–41/211–234	V42-T149	V154-N211
KCTD6	AF-Q8NC69	-	D12-D107	M114-K234
KCTD7	AF-Q96MP8	1–46/196–224	P50-G158	Y162-W289
KCTD8	AF-Q6ZWB6	1–41/148–203/325–434	E44-L145	R205-P322
KCTD9	AF-Q7L273	73–88	D89-S191	- ^4^
KCTD10	AF-Q9H3F6	1–18/274–295	Y33-Q129	P141-E260
KCTD11 ^2^	AF-Q693B1	-	G14-A123	A126-H271
KCTD12	AF-Q96CX2	1–29/132–204/	P34-A131	R206-E325
KCTD13	AF-Q8WZ19	1–40/272–303/311–329	K41-E142	I150-T271
KCTD14	AF-Q9BQ13	1–28	T33-D124	M125-W255
KCTD15	AF-Q96SI1	1–43/266–283	A56-R162	A165-E265
KCTD16	AF-Q68DU8	1–21/124–161/281–389	E25-T122	K162-P280
KCTD17	AF-Q8N5Z5	1–29/197–260/265–321	G30-V135	P139-H196
KCTD18	AF-Q6PI47	242–426	D12-S118	P140-L242
KCTD19	AF-Q17RG1	108–171/259–282/ 501–756/788–803	D13-E107/ V172-M258/ Q396-Q487 ^3^	- ^4^
KCD20	AF-Q7Z5Y7	1–115/359–419	E117-C216	D218-E356
KCTD21	AF-Q4G0X4	-	P4-K107	N109-R260
KCNRG	AF-Q8N5I3	246–272	E5- Q104	P107-I245
SH3KBP1	AF-Q8TBC3	139–192/604–707	E19-R118	- ^4^
TNFAIP1	AF-Q13829	1–27/258–290/299–316	K28-S130	I137-E257
BTBD10	AF-Q9BSF8	1–148/409–475	M149-C266	D268-W405

^1^ Confident regions correspond to those with AF parameter LDDT > 70. Unstructured regions correspond to long (>15 residues) fragments with a marginal content of secondary structure and a low confidence prediction (LDDT < 70). ^2^ The sequence numbering refers to the long variant. The structural data were derived from the model obtained with the Colab server (see Methods for details). ^3^ The three regions correspond to the three BTB domains of the protein. ^4^ The KCTD-CTD domain has been not clearly identified in the AF structure of the protein (see text).

**Table 3:** Dali Z-score similarity matrix derived from the structural alignment of protein C-terminal domains. The clade definition is the one used in Figure S1. Dashes are reported for pairs for which Dali does not detect any significant similarity. The names of the proteins are abbreviated due to space limitations.

		K1	K15	K8	K12	K16	K6	K11	K21	K4	K20	B10	K18	K19	KCN	K7	K14	K10	K13	TNF	K2	K5	K17	K3	SHK	K9
Clade A	**K1**	23.3	19.6	10.1	9.6	9.8	8.1	5.9	2.7	2.2	-	-	2.9	-	9.5	2.3	2.5	-	-	-	4.1	3.9	3.7	-	-	-
	**K15**		23.3	10.4	9.9	10.1	7.9	5.1	2.1	2.0	-	-	2.7	-	9.8	2.6	3.6	-	-	-	4.9	4.8	5.0	-	-	-
Clade F	**K8**			22.8	19.5	20.4	7.8	5.8	-	2.0	-	-	-	-	8.1	2.3	-	-	-	-	2.7	2.8	2.8	-	-	-
	**K12**				22.8	19.2	7.6	5.7	-	-	-	-	-	-	8.9	-	-	-	-	-	2.5	2.5	2.6	-	-	-
	**K16**					22.7	7.5	5.0	-	2.1	-	-	2.2	-	8.0	-	2.3	-	-	-	3.2	3.0	3.3	-	-	-
Clade B	**K6**						24.4	7.8	5.8	3.3	-	-	3.1	2.2	11.6	2.2	2.3	-	-	-	3.7	3.8	3.5	-	-	-
	**K11**							26.9	14.2	5.3	-	-	-	-	8.7	-	-	-	-	-	-	-	-	-	-	-
	**K21**								25.2	2.1	-	-	-	-	9.2	-	-	-	-	-	-	-	-	-	2.7	-
	**K4**									27.3	-	-	-	2.3	3.3	-	-	-	-	-	-	-	-	-	-	-
Clade G	**K20**										25.3	22.9	-	-	-	5.6	6.6	-	-	-	-	-	-	-	-	-
	**B10**											25.0	-	2.2	2.1	5.7	6.6	-	-	-	-	-	-	-	-	-
	**K18**												21.8	-	2.5	-	-	-	-	-	-	-	-	-	-	-
	**K19**													54.7	-	-	2.0	-	-	-	-	-	-	-	-	-
	**KCN**														27.0	3.8	3.4	-	-	-	4.8	4.5	4.8	-	-	-
	**K7**															24.9	13.4	-	-	-	3.0	3.0	-	-	-	-
	**K14**																27.1	2.4	2.2	2.4	3.0	3.0	3.0	-	-	-
Clade C	**K10**																	25.6	21.4	23.5	-	-	-	-	-	-
	**K13**																		27.0	22.4	-	-	-	-	-	-
	**TNF**																			26.7	-	-	-	-	-	-
Clade E	**K2**																				12.1	10.7	10.8	-	-	-
	**K5**																					12.0	10.2	-	-	-
	**K17**																						11.9	-	-	-
Clade D	**K3**																							62.7	50.3	-
	**SHK**																								63.6	-
	**K9**																									39.6

## Data Availability

All datasets analyzed in this study are available from the corresponding authors on reasonable request.

## Supplementary Materials

The following are available online at https://www.mdpi.com/article/10.3390/biom11121862/s1, Figure S1: Human KCTD protein tree, Figure S2: Schematic representation of AlphaFold KCTD19 structure, Figure S3: Schematic representation of PDB experimental structures for selected human KCTD proteins, Figure S4: Superimposition of different human KCTD1 structures, Figure S5: Similarity heatmap showing the relationships between the KCTD-CTD domains of the analyzed proteins, Figure S6: Superimposition of the C-terminal domains of AF models for SHKBP1 protein and the KCTD21 protein, Figure S7: Structural comparison of the AF-KCTD4 protein domains with the AF-KCTD19 BTB2 protein domain, Figure S8: Correspondence analysis plot.
