# Supplementary material for: AlphaFold-Predicted Structures of KCTD Proteins Unravel Previously Undetected Relationships among the Members of the Family
Source: Biomolecules. 2021 Dec 10;11(12):1862. doi: 10.3390/biom11121862 (PMC8699099; doi:10.3390/biom11121862)
Supplement: Supplementary file 1 [file biomolecules-11-01862-s001.zip › biomolecules-1478650-supplementary.pdf]

biomolecules

MDPI

Supplementary Materials

AlphaFold-Predicted Structures of KCTD Proteins Unravel Previously Undetected Relationships among the Members of the Family

Luciana Esposito [1,*], Nicole Balasco [1], Giovanni Smaldone [2], Rita Berisio [1], Alessia Ruggiero [1] and Luigi Vitagliano [1,*]

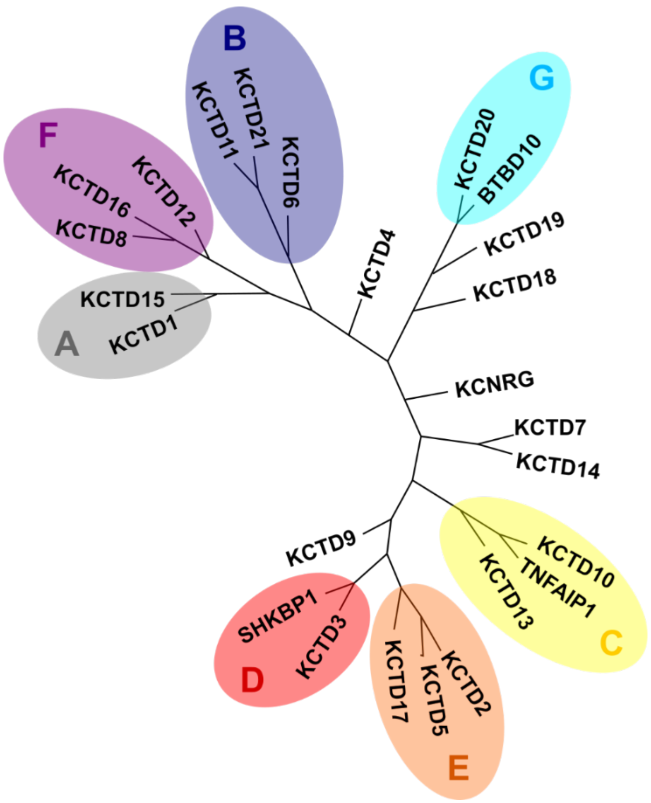

Figure S1. Human KCTD protein tree. The tree (adapted from Pinkas et al [15]) was based on the alignments of the sequences of the BTB domains by Skoblov et al [23]. Ovals represents the clades in which protein presenting sequence similarities in the C-terminal region were grouped.

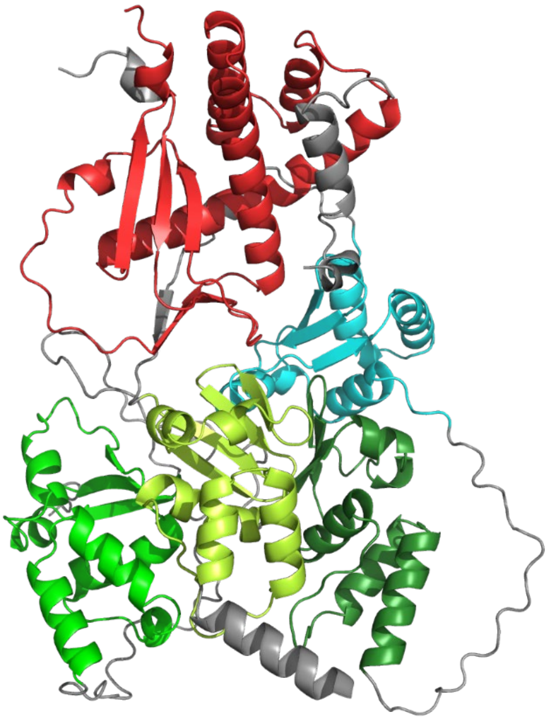

Figure S2. Schematic representation of AlphaFold KCTD19 structure. The BTB domains have been highlighted in green (BTB1), forest green (BTB2) and bitter lime (BTB3) (see Table 2 and Figure 2 for details).

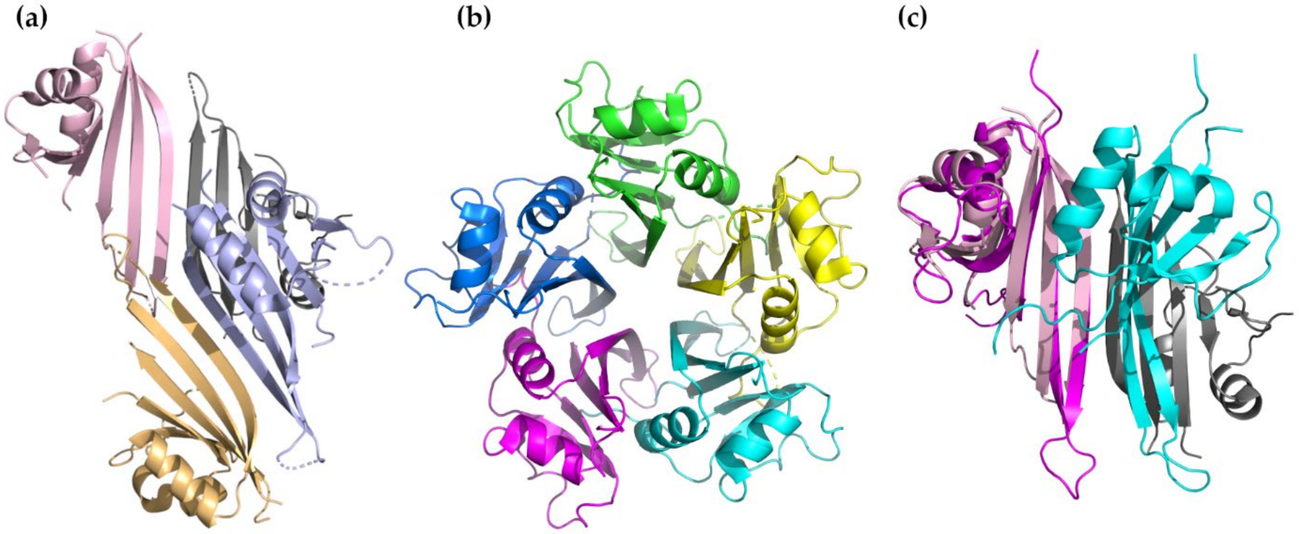

Figure S3. Schematic representation of PDB experimental structures for selected human KCTD proteins. (**a**) KCTD8 protein (PDB code 6G57); (**b**) KCTD16 protein (PDB code 6QB7); (**c**) Superposition of two chains of KCTD8 (light pink) and KCTD16 (magenta) proteins and the relative positions of their correspondent adjacent chains in the two structures. .

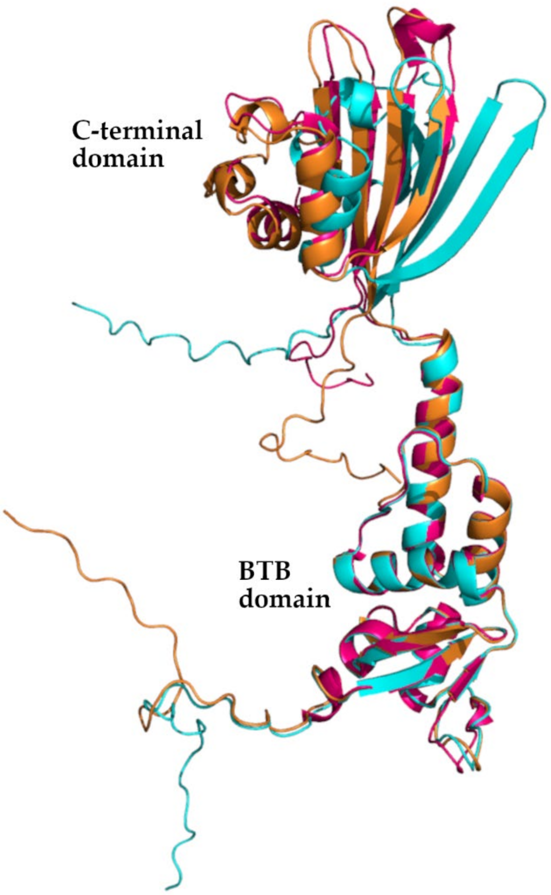

Figure S4. Superimposition of different human KCTD1 structures. The structural models have been aligned by superimposing the BTB domains. The AF model for KCTD1 is shown in orange; the Colab model is shown in cyan; the PDB experimental structure is shown in hot pink.

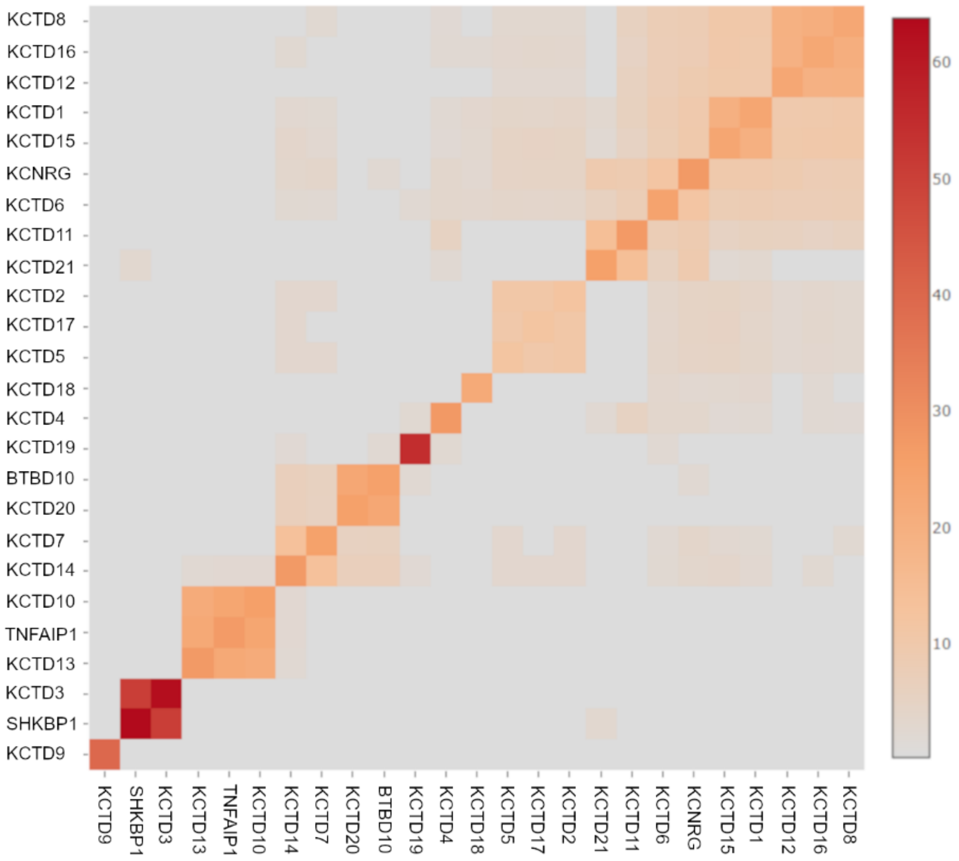

Figure S5. Similarity heatmap showing the relationships between the KCTD-CTD domains of the analyzed proteins. The color scale corresponds to the Dali Z-score values.

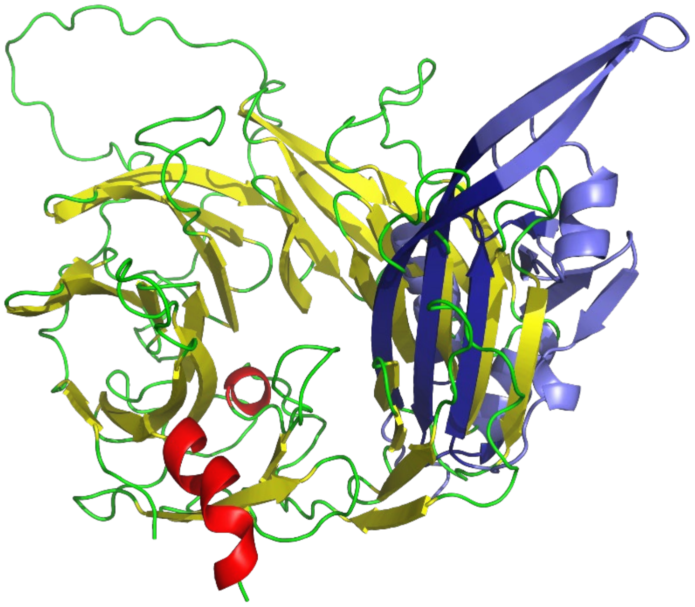

Figure S6. Superimposition of the C-terminal domains of AF models for SHKBP1 protein (with its secondary structure elements, sheet-helix-loop, depicted in yellow-red-green) and the KCTD21 protein (in blue, with the aligned regions highlighted in darker blue).

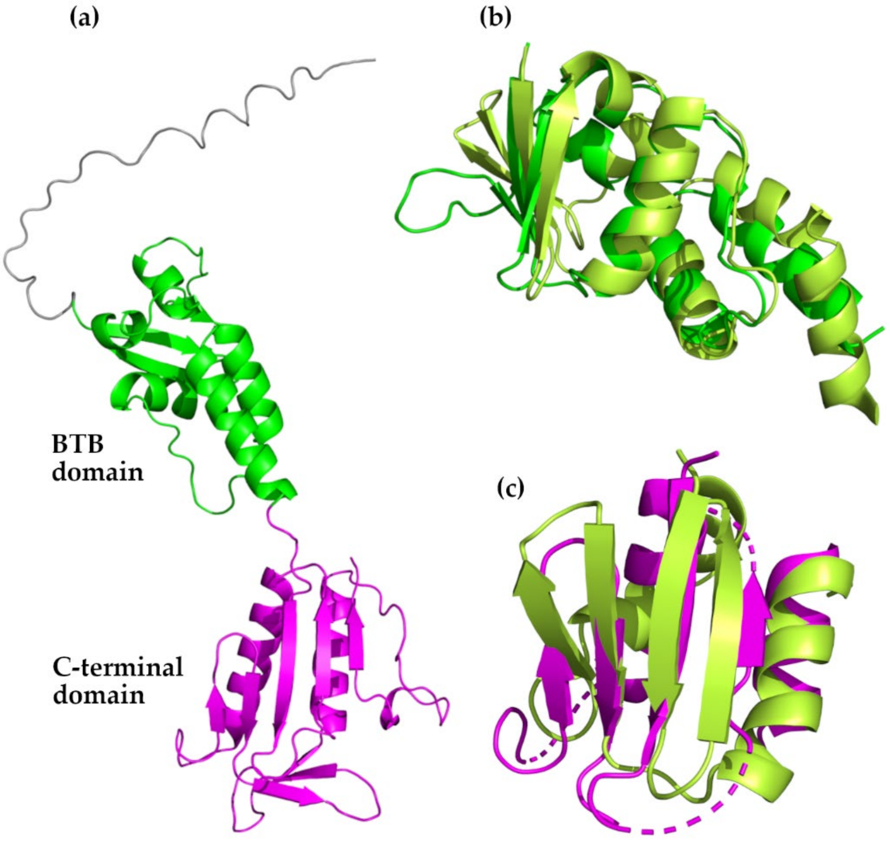

Figure S7. Structural comparison of the AF-KCTD4 protein domains with the AF-KCTD19 BTB2 protein domain. (**a**) AF-KCTD4 protein with its BTB and C-terminal domains highlighted in green and magenta, respectively; (**b**) Superposition of AF-KCTD4 BTB domain (green) and AF-KCTD19 BTB2 domain (bitter lime); (**c**) Superposition of AF-KCTD19 BTB2 domain (bitter lime) and the aligned regions of AF-KCTD4 C-terminal domain (magenta).

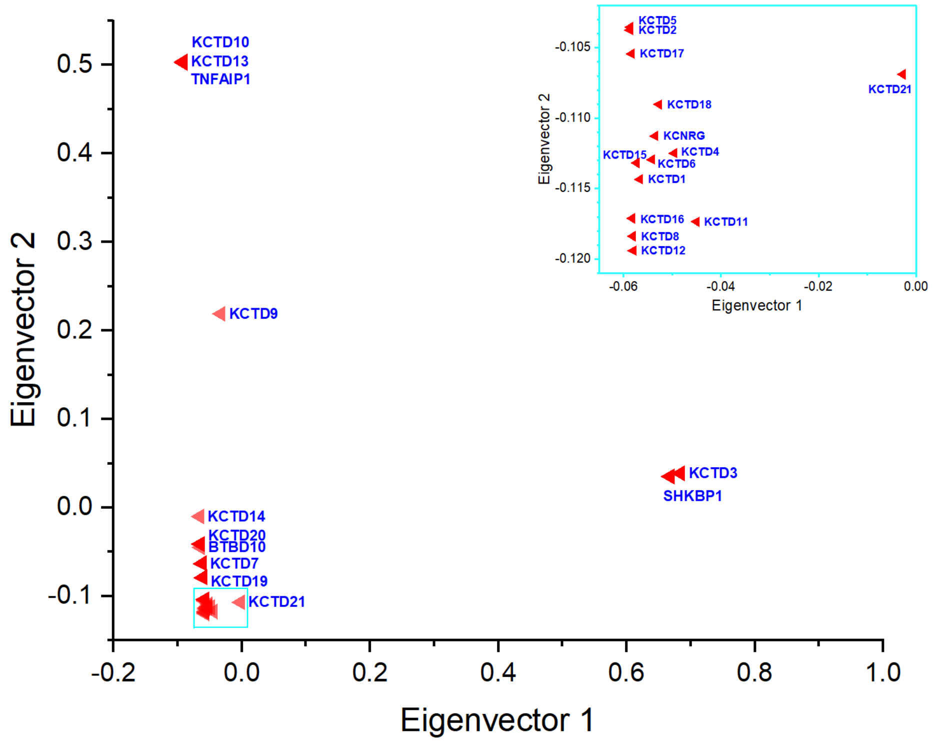

Figure S8. Correspondence analysis plot. Dali multidimensional scaling correspondence analysis of the Z-scores from the all-against-all comparison of AF C-terminal domains of KCTD structures. The results of the projection of the structures on the first two eigenvectors are plotted. The close-up view of the cyan region is shown in the inset on the top right.
